# Congenital Midureteric Stricture: Challenges in Diagnosis and Management

**DOI:** 10.1155/2015/969246

**Published:** 2015-04-02

**Authors:** Raashid Hamid, Nisar A. Bhat, Kumar Abdul Rashid

**Affiliations:** Department of Paediatric and Neonatal Surgery, Sher-i-Kashmir Institute of Medical Sciences (SKIMS), Soura, Srinagar, Jammu and Kashmir 190011, India

## Abstract

*Background*. Congenital midureteric stricture (MUS) is a rare malformation. We report our experience with five cases seen over a period of 4 years from 2010 to 2014. *Materials and Methods*. The study was based on the retrospective analysis of five patients diagnosed as having MUS. Diagnosis was suspected after fetal ultrasonography (USG) in one patient and magnetic resonance urography (MRU) in four patients. Retrograde pyelography (RGP) was performed on three patients. The final diagnosis was confirmed during surgical exploration in all the patients. *Results*. MRU was found to be a good investigation method. It showed the site of obstruction in the ureter in all instances. Intravenous urography detected proximal ureteric dilatation present in two of the patients. RGP delineates the level of stricture and the course of ureter, as shown in our cases. All patients had significant obstruction on the affected side. Four patients underwent ureteroureterostomy, all of whom had satisfactory results. In one patient, ureteric reimplantation was carried out due to distal small ureteric caliber. *Conclusion*. This rare entity is often misdiagnosed initially as pelviureteric junction obstruction. MRU is an excellent option for the anatomical location and functional assessment of the involved system. At the time of surgical correction of a ureteral obstruction, RGP is a useful adjunct for delineating the stricture level and morphology.

## 1. Introduction

Midureteric stricture (MUS) is a rare cause of hydronephrosis (HDN) in neonates and is often misdiagnosed as pelviureteric junction (PUJ) obstruction in the first instance. Accurate preoperative diagnosis with IVP and radionuclide scans may not be possible in all cases. Hence, additional investigations with magnetic resonance urography (MRU) and retrograde pyelography (RGP) are required to arrive at accurate diagnosis [1].

We report here our experience in clinical findings, radiological investigations, and operative treatment in five infants with MUS.

## 2. Case Presentation

This is a descriptive study based on the retrospective analysis of five cases diagnosed as having MUS during the period 2010–2014.

### 2.1. Case 1

A baby girl (5 months old) was diagnosed as having unilateral (Rt.) HDN on antenatal ultrasonography (USG). Postnatal USG scan showed a right HDN. IVP and DTPA scans confirmed the diagnosis of right PUJ obstruction. On exploration, MUS was diagnosed and ureteroureterostomy was performed over a double J (DJ) stent.

### 2.2. Case 2

An 8-month-old baby boy was referred to our department as a suspected PUJ obstruction on left side. IVP showed delayed drainage from the pelvicalyceal systems (PCS) along with the dilatation of upper ureter. Micturating cystourethrography (MCUG) was normal. MRU showed a transition in ureteral caliber at obstructed site. Cystoscopy and RGP were performed, which showed an MUS of approximately 2 cm.

### 2.3. Case 3

A 4-month-old baby boy was antenatally diagnosed as having right HDN. IVP showed dilated PCS with delayed drainage. MRU showed that upper ureter was also dilated with an abrupt change in caliber at midureter. The RGP done before surgery showed a stricture, which was managed by oblique ureteroureterostomy. The baby did well in follow-up.

### 2.4. Case 4

A 6-month-old baby boy was diagnosed as having crossed renal ectopia right to left with PUJ obstruction in the crossed moiety (left). MCUG was normal. IVU showed PCS dilatation in one renal moiety. MRU showed upper-ureteric dilatation and PCS of left crossed renal moiety. On exploration, a ureteric stricture of crossed renal moiety was diagnosed and the subjected ureter was reimplanted.

### 2.5. Case 5

A 5-month-old baby boy was diagnosed as having HDN on the left side on antenatal USG. MCUG was normal, and USG and IVP again showed hydroureteronephrosis (HUN). MRU was performed, which revealed ureteric obstruction. Exploration of the left ureter confirmed a tight stricture in midureter, which was excised, and end-to-end oblique anastomosis was carried out over a stent.

## 3. Results

**Figure 1:**
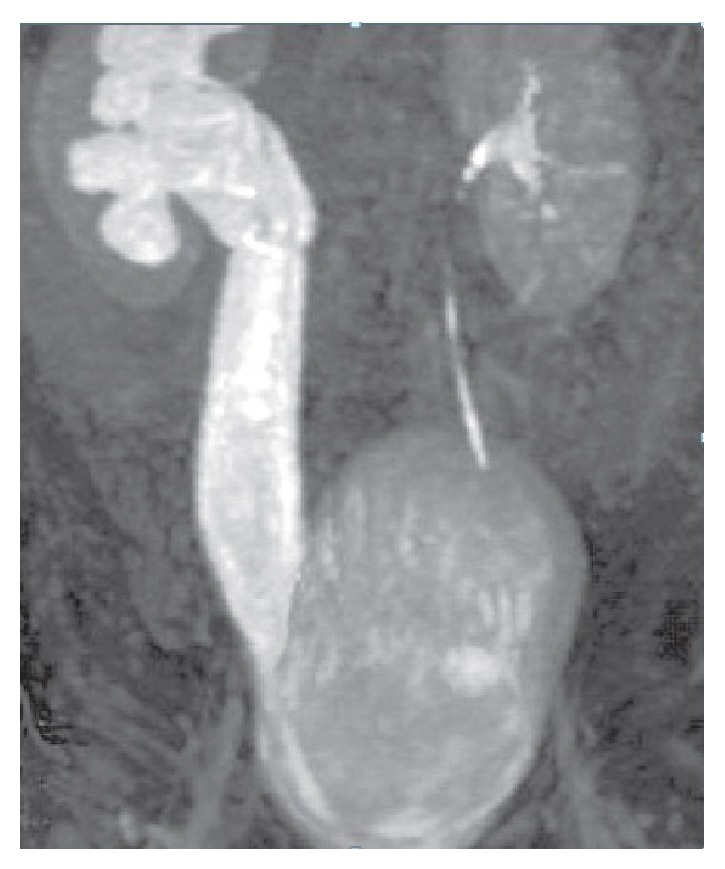
On the coronal MR urography image, the right ureter and the pelvicalyceal system are seen dilated.

**Figure 2:**
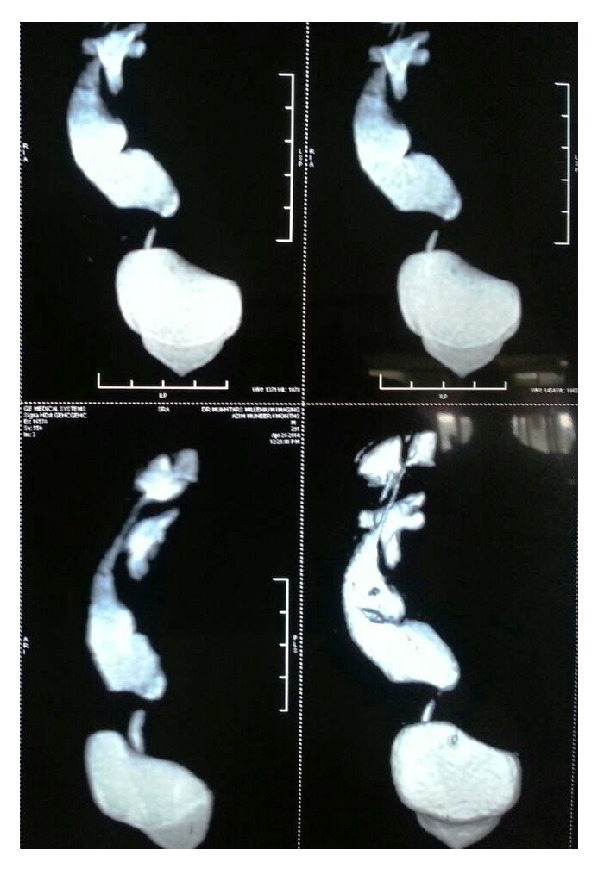
MRU showing a midureteric stricture in crossed ectopic left to right kidney.

**Figure 3:**
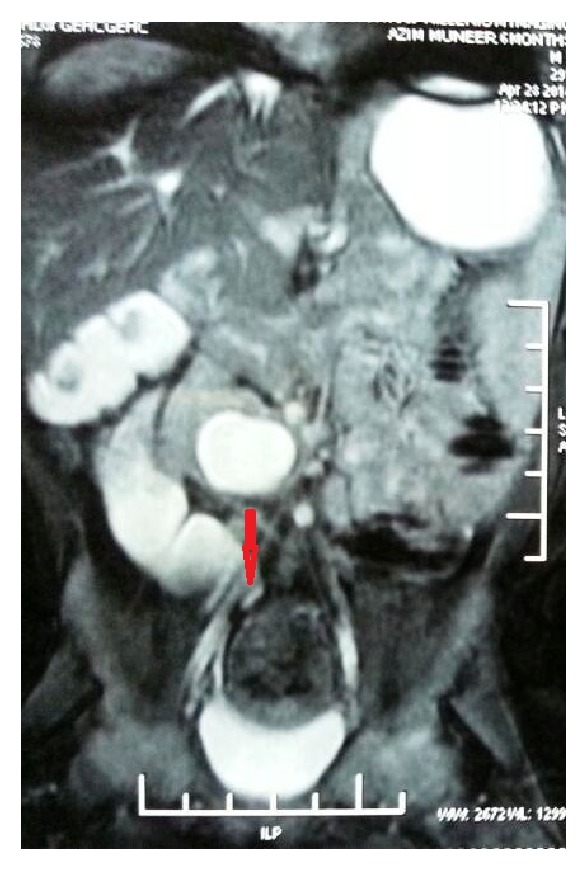
Red arrow shows a stricture site of left ureter.

Antenatal USG was available in three cases, and in only one case, ureteric stricture was suspected. Postnatal USG showed crossed renal ectopia in one patient (case 4) and ureter was found to be dilated on USG in cases 2 and 5. IVU was performed in all the five cases. IVU in three cases (cases 1, 3, and 4) showed a PUJ obstruction. The radionuclide scan provided information about differential function and obstructive pattern in all five cases. MRU was performed in four cases, which confirmed the diagnosis of ureteric stricture in these cases (Figure 1). Furthermore, MRU provided excellent anatomical details in case 5 with crossed renal ectopia (Figures 2 and 3).

The salient features and diagnostic findings in MUS in five patients are summarized in Table 1. All the patients were administered preoperative antibiotic therapy. In all the five cases, reflux was ruled out by MCUG.

As shown in our cases, USG does not always suggest the diagnosis of ureteric obstruction. IVU showed the HUN in cases 2 and 5. In the remaining three cases (cases 1, 4, and 3), IVU showed only dilatation of PCS. ^99m^Tc-DTPA scan was performed on all the five patients, which showed the differential function and degree of HDN and drainage.

MRU was performed on four patients (cases 2–5) and was more accurate than USG in assessing renal and ureteral anomaly. The ability of the magnetic resonance imaging to delineate the site of the ureteric obstruction corresponded to the intraoperative findings in all these four cases.

**Figure 4:**
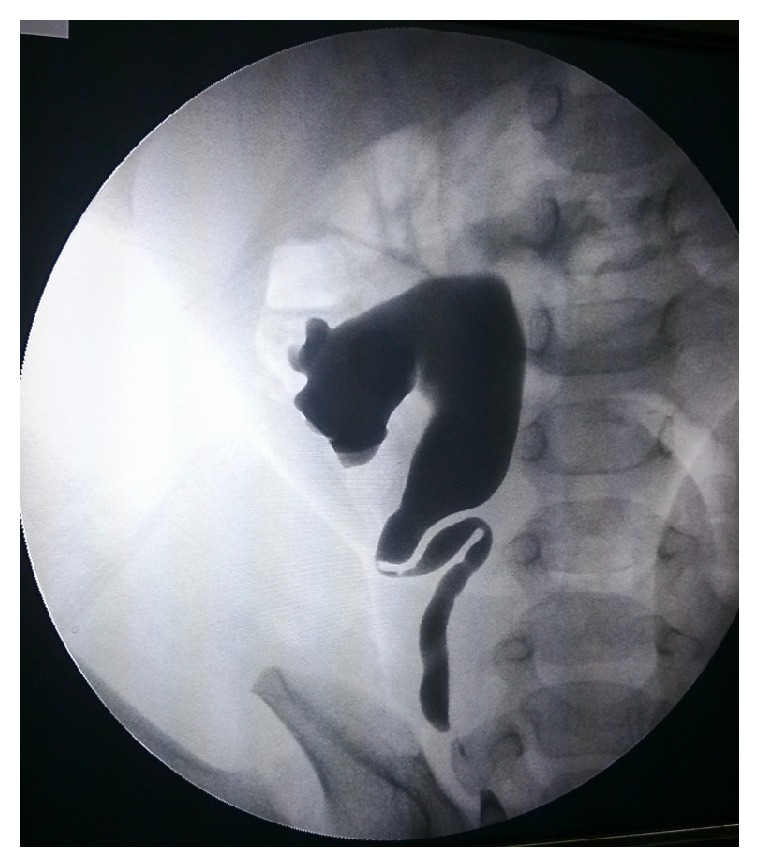
Retrograde pyelography demonstrating a midureteric stricture and proximal dilated ureter.

To add further details about the extent of the stricture, RGP was performed on three cases (cases 2, 3, and 5). It showed the site and length of the stricture. It also defined the caliber of the distal ureter (Figure 4).

**Figure 5:**
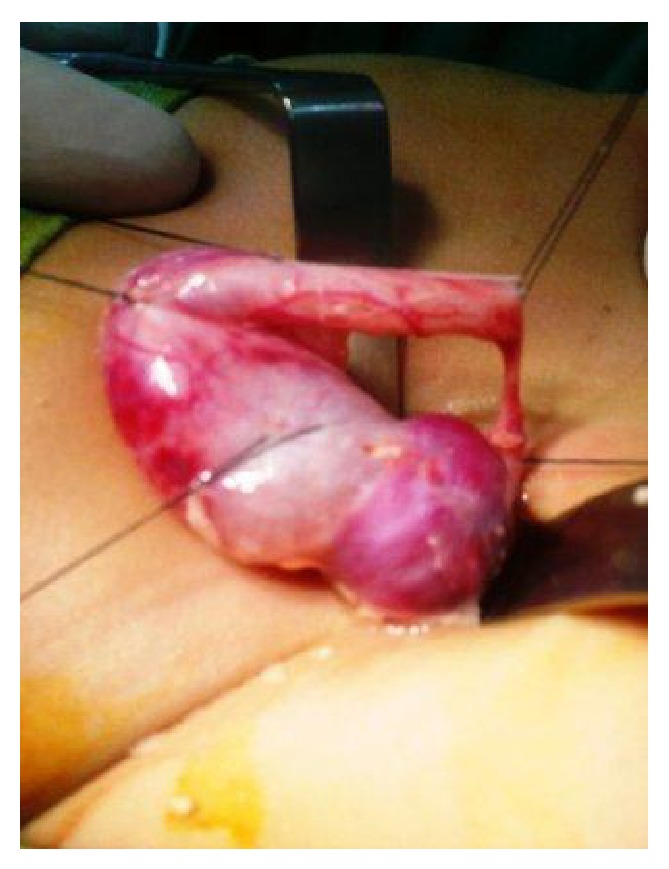
Photograph showing dilated ureter proximal to the stricture and distal normal caliber ureter.

**Figure 6:**
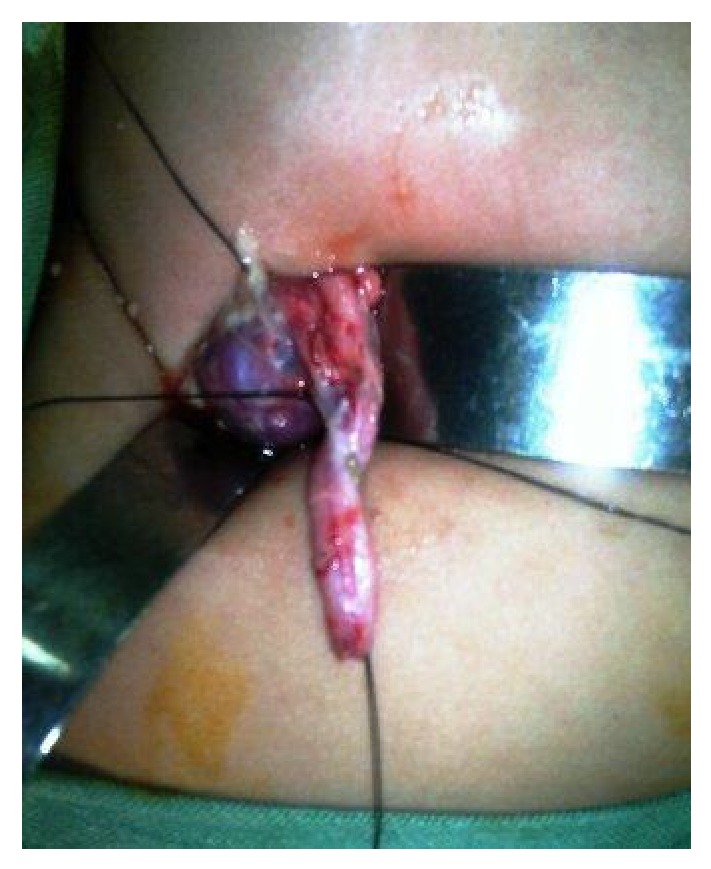
Photograph showing ureteric stricture opened longitudinally with no evident lumen.

The surgical management of ureteric stricture was ureteroureterostomy in 4 cases. Oblique anastomosis was performed in these four cases over a DJ stent. In one case, as the kidney was low and ectopic, the site of stricture was close to the bladder, and ureteric reimplantation was deemed more feasible (Figures 5 and 6).

Postoperatively, the stent was removed at 6 weeks. All the patients had uncomplicated postoperative course. Subsequent USG of renal tract showed improvement in HDN. Histopathological examination of the resected strictures showed subepithelial fibrosis.

## 4. Discussion

The cause of congenital ureteric stenosis (CUS) is not certain. Simple narrowing probably results from a disturbance in embryogenesis around the 11th or the 12th week with disturbances in development of mesenchyme contributing to ureteral musculature. CUS have been attributed to incomplete recanalization of the ureters [2]. Ureteric obstruction presents as HDN and upper-tract dilatation similar to that caused by PUJ obstruction, primary megaureter, and vesicoureteral reflux (VUR) and is often misdiagnosed. It may present as HDN in prenatal period [3]. With widespread use of prenatal ultrasonography, an increased number of children investigated for antenatal hydronephrosis are found to have congenital midureteral strictures. Two of the cases presented as antenatal HDN in our series. USG subsequently performed showed HDN in all five cases and ureteric dilatation in two cases. IVU showed HDN in all cases, but ureteric dilatation was shown in only two cases. The literature indicates that IVU is not always accurate [1], although the stricture was located accurately in two of our patients. MRU offers the combination of high-resolution anatomic imaging and functional information of the ureter and kidneys. MRU evaluates the urinary tract dilatation and differentiates it from obstruction [4, 5]. As shown in our cases, ureteral obstruction was diagnosed in four of cases on MRU. MRU unlike IVP and scan visualizes the ureters independent of the renal function [6]. MRU permits HDN and transition in ureteral caliber to be reliably detected even in nonfunctioning ureterorenal units. These findings could not be appreciated on either USG or diuretic renal scintigraphy. MRU was more accurate than USG and IVU in assessing ureterorenal anatomy, as shown in our cases. It has the potential to provide functional imaging comparable with diuretic renal scintigraphy. Some clinicians advocate this method to replace USG and diuretic renal scintigraphy in evaluation of HDN [7].

RGP, at the time of surgical correction of a presumed ureteral obstruction, is the most useful modality in diagnosing congenital ureteric stricture because it makes it possible to visualize the stricture [7]. Information provided by RGP facilitates the choice of surgical approach to the affected ureter.

We recommend the RGP should be performed in cases where information provided by USG, IVP, and diuretic renal scintigraphy is equivocal. Otherwise, MRU has the potential to give accurate anatomical, functional details of ureterorenal system and, arguably, the location of the stricture.

CUS can be associated with other renal abnormalities including solitary kidney (10 of John), contralateral blind ending ureter [8], and MUS in an ectopic ureter of a duplex system [9]. In one of our patients, stricture was present in the ureter of the crossed ectopic renal moiety. The presence of a solitary kidney makes early management of the stricture mandatory.

Cussen analyzed 124 obstructed ureters, stricture was found in 81 specimens [10]. Strictures are characterized by 60% decrease in luminal diameter and decrease in smooth muscle cells. Smooth muscle may be replaced by fibrous tissue, which we found in our four cases. Histopathological studies of the stenotic zone showed normal transitional epithelium and diminished population of normal-appearing smooth muscle cells.

Endourological dilatation or endoscopic incision of the ureteral stricture may be considered, but these techniques have a lower chance of success than a ureteroureterostomy or ureteral reimplant [11]. Similar to the retrocaval ureter, treatment may require excision of redundant or kinked segments of the ureter. Open procedures with end-to-end anastomosis over DJ stent may be undertaken [12]. Endourological procedure is an alternative. At times, mobilization of the kidney may be required for tension-free primary anastomosis. If stricture is of several centimeters, it may be best to cut the segment in the middle and then spatulate the narrowed segment in both directions to avoid tension on anastomosis [13].

## 5. Conclusion

To conclude, MUS is rare but should be suspected if HDN is associated with upper-ureteral dilatation and normal MCUG. The goal should be to arrive at an appropriate preoperative diagnosis for better operative planning. Whenever conventional diagnostic methods are less informative, MR pyelography and RGP should be added to the diagnostic armamentarium. We recommend postnatal MRU in case the diuretic renal scintigraphy/IVU is less informative, to detect the site of narrowing. Tension-free primary ureteroureterostomy is the most favorable treatment option.

## Figures and Tables

**Table 1:** Salient clinical features and findings on different diagnostic tools in patients with midureteric stricture.

Case number	Presentation	Age/sex	Ultrasonography	IVU	Differential function of affected side	MRU	Retrograde pyelography	Operation
1	UTI	5 months/female	Antenatal USG-right side, grade 2 HDN	Dilated PCS with impaired drainage—PUJ	34%	—	—	Ureter-ureteric anastomosis over D-J stent

2	UTI	8 months/male	Left side, grade 3 HDN with upper ureteric dilatation	Delayed drainage from PCS with dilated upper ureter	35%	Abrupt ureteral narrowing at obstructed area—dilated PCS and proximal ureter	Confirmed on RGP	Ureter-ureteric anastomosis over D-J stent

3	Antenatal hydronephrosis on right side	4 months/male	Right side, grade 2 HDN	Dilated PCS with impaired drainage—PUJ	35%	Delineates the site of obstruction Proximal dilatation of ureter and PCS	Confirmed on RGP	Ureter-ureteric anastomosis over D-J stent

4	—	6 months/male	Crossed left to right ectopia, grade 2 HDN	Two renal units on right side, with HDN in one unit	27%	Crossed left to right ectopia with—dilated ureter and pelvis of left renal unit	—	Ureteric reimplantation

5	Antenatal hydronephrosis	5 months/male	Left side, grade 2 HDN with ureteric dilatation	Grade 3 hydroureteronephrosis	28%	MRU revealed midureteric obstruction	Confirmed on RGP-	Ureter-ureteric anastomosis over D-J stent
